# Morphological evidence for neuronal connections between the olfactory neurogenic region and the striatum in adult rats

**DOI:** 10.3389/fncir.2025.1605961

**Published:** 2025-09-17

**Authors:** Kamila Fabianová, Marcela Martončíková, Ivo Vanický, Juraj Blaško, Alexandra Popovičová, Monika Žideková, Enikő Račeková

**Affiliations:** Department of Regenerative Medicine and Cell Therapy, Institute of Neurobiology, Biomedical Research Center, Slovak Academy of Sciences, Bratislava, Slovakia

**Keywords:** postnatal neurogenesis, Fluoro-Gold, subventricular zone, rostral migratory stream, neural circuit, nitric oxide, secretagogin

## Abstract

The regulatory mechanisms of postnatal neurogenesis in the subventricular zone (SVZ) and the rostral migratory stream (RMS) are still not fully understood. Recent evidence suggests that neurogenesis in the SVZ/RMS may be regulated by neurons located directly in these regions. To date, two populations of neurons residing in the SVZ/RMS, which display the morphological characteristics of mature neurons, have been identified: nitric oxide (NO)-producing neurons and neurons expressing secretagogin (SCGN). The aim of our study was to map the possible projections of these neuronal populations in the SVZ/RMS. All experiments were performed on adult male Wistar albino rats. To test the hypothesis that nNOS- and SCGN-positive neurons of the SVZ and RMS send their axons to the striatum, we injected this target brain structure with the retrograde fluorescent tracer Fluoro-Gold (F-G). To verify the identity of nitrergic neurons and SCGN- expressing neurons, double immunofluorescent labeling with anti-nNOS/anti-SCGN and anti-F-G was performed. Microscopic analysis revealed the presence of F-G, administered into the striatum, in cells of the SVZ and different parts of the RMS. F-G-labeled cells in the SVZ/RMS were identified as either nitrergic neurons or SCGN-expressing neurons. Our results demonstrate a connection between mature neurons of the SVZ/RMS and the striatum.

## 1 Introduction

The main neurogenic region in the adult mammalian brain is the subventricular zone (SVZ) of the brain lateral ventricle. Precursor cells of the SVZ migrate through a well-defined pathway, known as the rostral migratory stream (RMS), toward their final destination, the olfactory bulb (OB) (Ming and Song, 2005). In adult rodents, the RMS is an L-shaped structure that can be divided along the caudal-rostral axis into three topographically consecutive anatomical parts: the vertical arm, the elbow, and the horizontal arm (Peretto et al., 1999; Raceková et al., 2005).

Postnatal neurogenesis in the SVZ-RMS-OB is a complex process that involves different phases of neuronal maturation. During their migration via the RMS, neuroblasts proliferate, and in the OB, they differentiate into inhibitory interneurons and integrate into existing neural circuits (Akter et al., 2021). New interneurons are also added to the accessory olfactory bulb (AOB) (Bonfanti et al., 1997), which resides in the dorsal-posterior region of the OB and is known to process olfactory information related to sexual behavior (Yoo et al., 2017).

Despite significant advances, the regulatory mechanisms of adult neurogenesis in the SVZ-RMS-OB are still not fully understood. Various molecular players have been found to regulate specific stages of adult neurogenesis in mammals. Different morphogens, growth factors, and transcription factors play critical roles in this process (Liu and Song, 2016). Numerous neurotransmitters, such as serotonine, dopamine, glutamate, GABA (Platel et al., 2010) and nitric oxide (NO), a free radical signaling molecule (Gutiérrez-Mecinas et al., 2007) have also been shown to regulate adult neurogenesis.

Moreover, recent findings suggest that adult neurogenesis could also be regulated by neural circuits. This suggestion is based on the detection of mature neurons directly in the SVZ-RMS (Raceková et al., 2005; Paez-Gonzalez et al., 2014; Hanics et al., 2017). To date, two cell populations displaying the morphological characteristic of mature neurons residing within the RMS have been identified: NO-producing neurons (Raceková et al., 2005) and neurons expressing secretagogin (SCGN) (Hanics et al., 2017). In addition, another population of neurons has been described in the SVZ – a small group of cholinergic neurons (ChAT + neurons) that reside in the subependymal space adjacent to the lateral ventricle (Paez-Gonzalez et al., 2014).

In our laboratory, we have previously demonstrated that NO-producing neurons are predominantly located in the vertical arm of the RMS, which emerges directly from the SVZ and extends between the striatum and corpus callosum (CC) (Blasko et al., 2013). We have also showed that the long processes of NO-producing neurons in the RMS extend deeply into surrounding brain structures, particularly the striatum (Raceková et al., 2005). SCGN-producing neurons are primarily found in the outer region of the adult rat RMS, significantly outnumbering those in its inner compartment (Hanics et al., 2017).

A necessary criterion for proposing the neuronal regulation of postnatal neurogenesis in the SVZ/RMS region is the identification of neuronal connections of NO-producing and SCGN-producing neurons. Recently, a novel neural circuit of ChAT + neurons located in the SVZ has been identified (Paez-Gonzalez et al., 2014). These neurons receive inputs from a specific calretinin-positive neuronal population located in the anterior cingulate cortex (Naffaa et al., 2021). However, it remains unknown whether the populations of NO- and SCGN-producing neurons residing within the SVZ/RMS region are also connected with other brain structures.

Based on previous morphological findings regarding the localization of nitrergic (Raceková et al., 2005) and SCGN-expressing SVZ/RMS neurons (Hanics et al., 2017), as well as the anatomical proximity of the SVZ/RMS to the striatum, we hypothesized that these neurons are likely connected to this structure. A specific neuronal connection between the striatum and the SVZ has previously been identified (Young et al., 2014). The authors have shown that GABAergic neurons in the striatum extend both dendrites and axons into the SVZ, establishing functional connections. Additionally, later work revealed that NO-containing GABAergic striatal neurons occasionally send nitrergic axons into the SVZ intermingled with chains of neuroblasts (Moreno-López et al., 2000).

In order to determine whether mature neurons (NO and/or SCGN+) in the SVZ-RMS send their axons to the striatum, we injected the retrograde fluorescent tracer Fluoro-Gold (F-G) into this brain structure. We demonstrated that F-G, when administered into the striatum, labeled the cells residing in the SVZ and all parts of the RMS. Using double immunohistochemistry, we identified F-G + cells in the SVZ/RMS as nNOS+ or SCGN + cells. Taken together, this study provides morphological evidence of connections between mature SVZ/RMS neurons and the striatum.

## 2 Materials and methods

### 2.1 Animal model

All experiments were performed on 14 adult (3 months old) male albino Wistar rats (bred at a certified vivarium of the Institute of Neurobiology BMC SAS imported from Velaz, Czech Republic) weighing 290–330 g. The experiments were performed in accordance with a protocol for animal care, which was ratified by the European Communities Council Directive (2010/63/EU) and with approval of the State Veterinary and Food Administration of the Slovak Republic under the supervision of the Ethical Council of the Institute of Neurobiology BMC SAS.

### 2.2 Experimental design

To reveal potential connection of NO+ and SCGN + neurons located in the SVZ/RMS with striatal neurons, a retrograde fluorescent tracer Fluoro-Gold (F-G; Fluorochrome, LLC, USA) was used. F-G was chosen for its unique properties, including intense fluorescence, high resistance to fading, and no diffusion from labeled cells. It is also ideal for use in combination with other fluorophores in multiple labeling studies (Schmued and Fallon, 1986; Schmued, 2016). The tracer F-G is a widely used marker for mapping neuronal pathways in various structures of the CNS as well as in peripheral nerves (Merchenthaler, 1991; Yu et al., 2015). F-G can be administered to a discrete brain region *in vivo* and it reveals neuronal connectivity via axonal transport of the tracer (Schmued, 2016). It appears yellow under UV illumination and is ideal for combining with other red or green fluorophores in multiple labeling studies (Schmued, 2016). In addition, for better visualization of the F-G, specific antibodies against F-G have been developed.

In this study, rats were anesthetized, prepared for stereotactic surgery, and fixed in a stereotactic headholder. The injection sites were located on the rat skull using stereotactic equipment (the injection coordinates were ML-1.4 mm, AP-1.8 mm, DV-5.5 mm from bregma, Supplementary Figure 1). A small incision was made on the scalp at the injection site using a scalpel. Rats received pressure injections of F-G (0.5 μl; 2% F-G, diluted in 0.9% saline) delivered via a Hamilton syringe into the striatum. After administration of the marker, the surgical wound was sutured and the rats were allowed to survive for 5 days.

In a pilot experiment, we aimed to visualize the injection site and assess the primary distribution of the applied F-G. For this purpose, two rats were given bilateral injections of F-G. Twenty-four hours after the injections, the animals were perfusion fixed, and their brains were sectioned coronally. In a series of sections, we have confirmed that the F-G was localized exclusively in the striatum, where it had diffused and formed a circular patch around the injection site (Supplementary Figure 2).

### 2.3 Tissue processing

After survival time the animals were deeply anesthetized with isoflurane and i.p. administration of chloral hydrate, followed by transcardial perfusion with 4% paraformaldehyde in 0.1 M phosphate buffer (PB). The brains were removed from the skulls. After overnight postfixation in 4% PFA in 0.1 M PB, the brains were placed in 30% sucrose in 0.1 M PB for cryoprotection. Sagittal and coronal sections, 30 μm thick, were then cut from the right hemispheres using a cryostat and collected in dishes containing 0.1 M phosphate-buffered saline (PBS). The sections were processed by immunohistochemical method. Anti-Fluoro-Gold antibody was used to enhance the F-G signal according to the standard protocol for F-G immunohistochemistry (Akhavan et al., 2006). The sections were double labeled with anti-Fluoro-Gold + anti-nNOS or anti-Fluoro-Gold + anti-SCGN antibodies to verify the identity of F-G + neurons.

### 2.4 Immunohistochemistry

The sections were washed three times in 0.1 M PBS for 10 min at room temperature (RT) and then incubated with a blocking solution, 10% normal goat serum (NGS) in PBS containing 0.3% Triton X-100 (PBS-T) for 1 h at RT. Then, the sections were incubated with primary antibodies diluted in 1% blocking buffer (NGS in PBS-T) for 48 h at 4°C. After washing with PBS, the sections were incubated in corresponding secondary antibodies diluted in 1% blocking buffer (NGS in PBS-T) for 1 h at RT. After the incubation, the sections were washed three times in PBS and mounted in Vectashield with DAPI (Vector Laboratories, Burlingame, USA) or in ProLong with DAPI (Cell signaling technology, Massachusetts, USA). The following primary antibodies were used: rabbit anti-Fluorescent Gold primary antibody (1:500, Merck, USA), mouse monoclonal anti-nNOS antibody (1:100, ThermoFischer, USA), mouse anti-SCGN primary antibody (1:200, Santa Cruz Biotechnology, USA), guinea pig anti-synaptophysin (1:200, Alomone Labs, Israel). The corresponding secondary antibodies were used: rhodamine-conjugated goat anti-rabbit IgG (1:200, Abcam, Cambridge, UK), rhodamine-conjugated goat anti-mouse IgG (1:200, Abcam, Cambridge, UK), fluoresceine-conjugated goat anti-mouse IgG (1:200, Abcam, Cambridge, UK), AMCA-conjugated goat anti-mouse IgG (1:200, Abcam, Cambridge, UK), rhodamine-conjugated goat anti-guinea pig IgG (1:200, Abcam, Cambridge, UK).

### 2.5 Microscopic and quantitative analysis

Sections were examined under a fluorescence microscope (Olympus BX-51 fitted with an Olympus DP50 digital camera system) at magnification × 40 and confocal laser scanning microscope (Leica TCS SPE, Leica, Mannheim, Germany) at magnification × 400. Images were acquired with Quick Photo Micro 2.3 software and arranged in PowerPoint.

For quantitative analysis of cells labeled with F-G, nNOS and SCGN in the SVZ/RMS, images of sagittal brain sections were taken at magnification 40×. The sections on which the SVZ and the entire extent of the RMS were visible (6–7 per animal) were used for the analysis. The labeled cells were counted along the whole extent of the SVZ and in the vertical arm of the RMS. GraphPad Prism 5.0 (GraphPad Software Inc., USA) was employed for statistical analysis of all data. Results were expressed as mean ± standard error of the mean (SEM).

For the quantification of neuronal morphology, the measurement of SCGN+ and nNOS + cells in the SVZ/RMS was carried out using ImageJ version 1.51 software (Bethesda, Maryland, USA). Cell profiles were manually outlined and the digital images of the cell silhouettes were then used as input for automatic extraction of cell dimensions. The Feret’s diameter of SCGN+ and nNOS + cells were expressed as mean ± SEM.

## 3 Results

### 3.1 F- G-positivity in the SVZ/RMS

**FIGURE 1:**
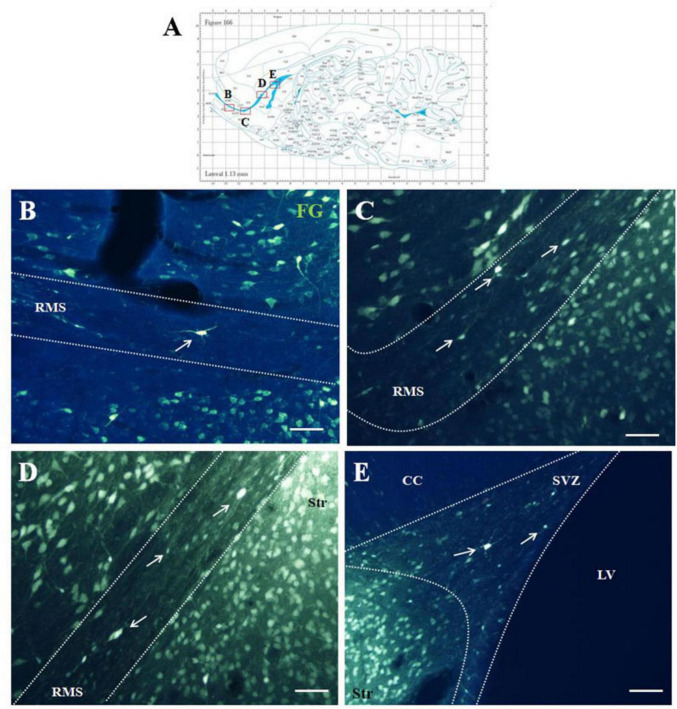
F-G-positivity in the SVZ/RMS on sagittal sections. Diagram of sagittal brain section from the rat brain atlas was adapted from Paxinos and Watson (2013). Red squares indicate the areas of the photomicrographs that are below **(A)**. Photomicrographs showing the distribution of F-G-positive cells (indicated by arrows) in the SVZ **(B)** and in the vertical arm **(C)**, in the elbow **(D)** and in the horizontal arm **(E)** of the RMS. The SVZ and the RMS are demarcated by dotted lines. Scale bar 25 μm. CC, corpus callosum; RMS, rostral migratory stream; Str, striatum; SVZ, subventricular zone; LV, lateral ventricle.

**FIGURE 2:**
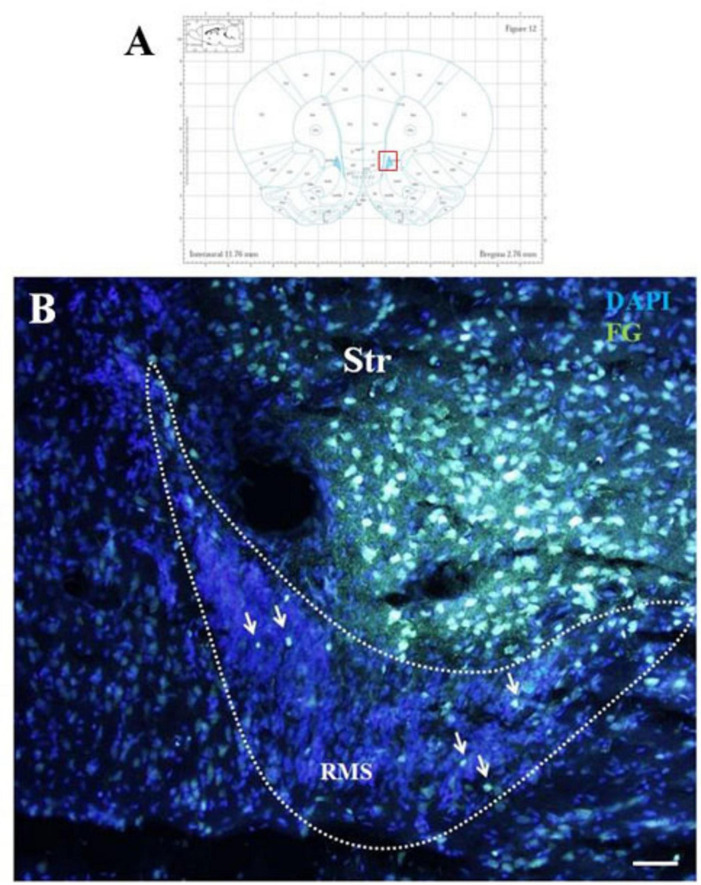
F-G-positivity in the RMS on coronal section. Diagram of coronal brain section from the rat brain atlas adapted from Paxinos and Watson (2013). Red square indicates the area of the photomicrograph that is below **(A)**. Photomicrograph showing the distribution of F-G-positive cells (indicated by arrows) in the RMS vertical arm **(B)**. The RMS is demarcated by dotted lines. Scale bar 25 μm. RMS, rostral migratory stream; Str, striatum.

Five days after the administration of the retrograde tracer Fluoro-Gold (F-G) into the striatum, F-G-positive cells were found in all parts of olfactory neurogenic area (Figure 1A). On sagittal sections F-G-positive cells were present within the SVZ (Figure 1B) as well as in all anatomical parts of the RMS, i.e., in the vertical arm (Figure 1C), the elbow (Figure 1D) and the horizontal arm (Figure 1E) of the RMS. F-G-positive cells within the RMS were visible also on coronal sections (Figure 2).

### 3.2 Identity of F-G-positive cells in the SVZ/RMS

To identify F-G-positive cells in the olfactory neurogenic area, we used immunoflourescent labeling with antibodies against known types of neurons residing in the SVZ-RMS. Anti-nNOS and anti-SCGN antibodies were used to label nitrergic and SCGN-producing cells, respectively.

#### 3.2.1 Double F-G/nNOS immunolabelled cells in the SVZ/RMS

**FIGURE 3:**
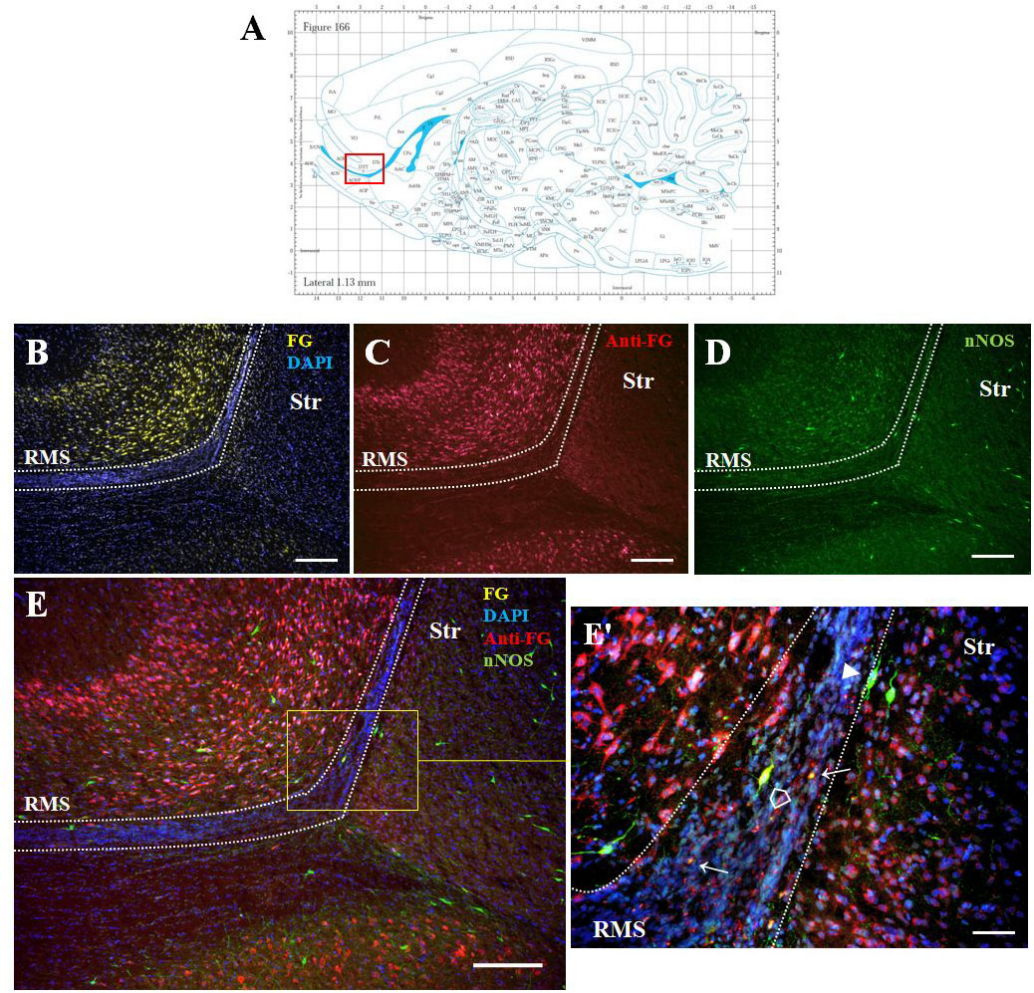
The identity of F-G-positive cells. Diagram of sagittal brain section from the rat brain atlas was adapted from Paxinos and Watson (2013). The red square indicates the areas of the photomicrographs that are below **(A)**. Distribution of F-G-labeled (yellow), anti-F-G-positive (red) and nNOS-positive (green) cells within the RMS **(B–E)** of adult rat. **(E’)** Magnification of the boxed area in panel **(E)**. Cell nuclei counterstained with DAPI (blue). The RMS is demarcated by dotted lines. Scale bar: **(B–E)** 100 μm, **(E’)** 50 μm. Open arrowheads (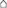): anti-F-G-positive + nNOS-positive cells; arrowheads (▶): anti-F-G-negative + nNOS-positive cells; arrows (→): anti-F-G-positive + nNOS-negative cells. RMS, rostral migratory stream; Str, striatum.

Double F-G/nNOS immunohistochemistry on sagittal sections (Figure 3) showed co-localization of both markers in some cells of the SVZ and RMS (Figures 3B–3E’). We noticed that not all F-G positive cells within the RMS were co-localized with nNOS (Figures 3E, E’). On the other hand, nNOS positive cells were present at the RMS outside margin. These cells were similar to those found within the migratory stream, but did not display F-G positivity (Figures 3E, E’).

**FIGURE 4:**
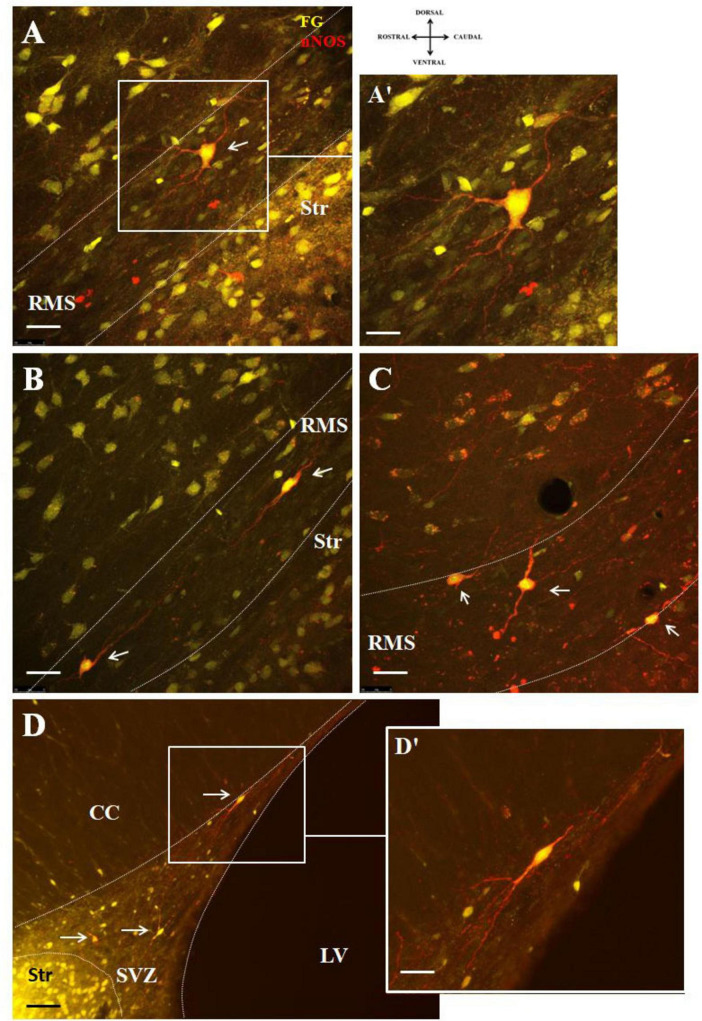
Representative confocal photomicrographs illustrating the different morphology of F-G-positive/nNOS-positive cells in the SVZ/RMS. nNOS-positive neurons (red) co-labeled with F-G (yellow) **(A,B)** in the vertical arm of the RMS, **(C)** in the elbow of the RMS and **(D)** in the SVZ (indicated by arrows). The SVZ and the RMS are demarcated by dotted lines. **(A’,D’)** Magnifications of the boxed areas in panels **(A,D)**. Scale bar: **(A–D)** 25 μm, **(A’,D’)** 10 μm. Str, striatum; CC, corpus callosum; LV, lateral ventricle; RMS, rostral migratory stream; SVZ, subventricular zone.

F-G/nNOS double labeled cells within the RMS were morphologically heterogenous. Multipolar cells were predominantly observed in the vertical arm of the RMS (Figures 4A, A’), while bipolar cells were distributed throughout the entire length of the RMS (Figures 4B, C) as well as in the SVZ (Figures 4D, D’). Bipolar cells accounted for approximately 64% of all labeled cells. F-G labeled nitrergic neurons were characteristic by abundant varicose processes in both the RMS and SVZ (Figure 4).

**FIGURE 5:**
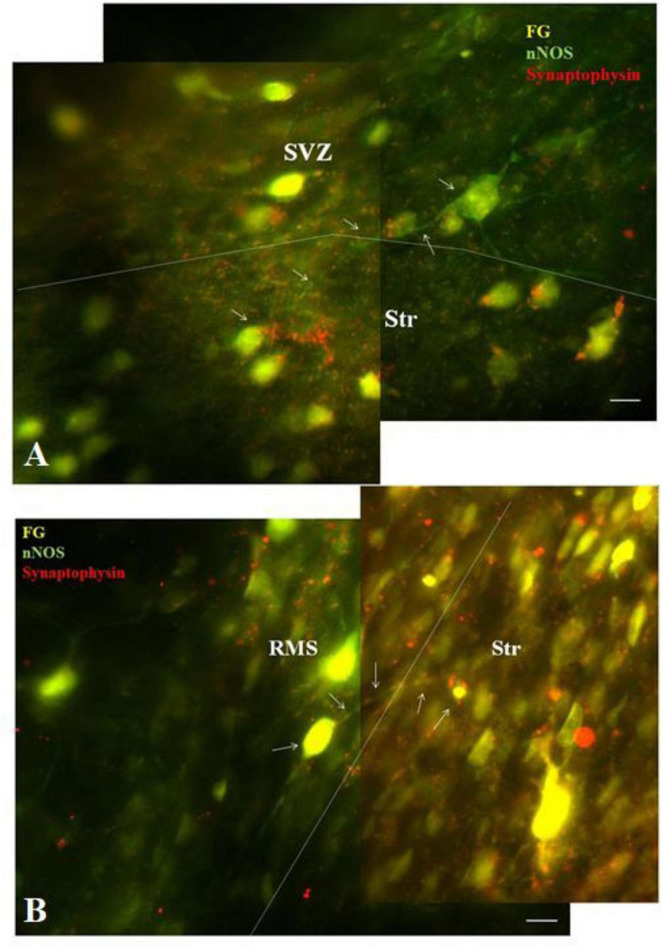
Co-labeling of F-G labeled nNOS + neurons with synaptophysin. Double-labeling of nNOS and synaptophysin showing synaptophysin-positive dots (red) at the projection terminals of nNOS positive (green) F-G labeled (yellow) cells of the SVZ **(A)** and the RMS **(B)** in the striatum. The borders between the SVZ/RMS and the striatum are indicated by dotted lines. Arrows indicate F-G+/nNOS+ neurons with synaptic vesicles. SVZ, subventricular zone; RMS, rostral migratory stream; Str, striatum. Scale bar 10 μm.

To demonstrate connectivity of F-G/nNOS-positive neurons in the SVZ and RMS with the striatum, co-labeling with synaptic marker, synaptophysin, was used. Our results revealed synaptophysin positive punctae at the projection terminals in the striatum (Figure 5).

#### 3.2.2 Double F-G/SCGN immunolabeled cells in the SVZ/RMS

**FIGURE 6:**
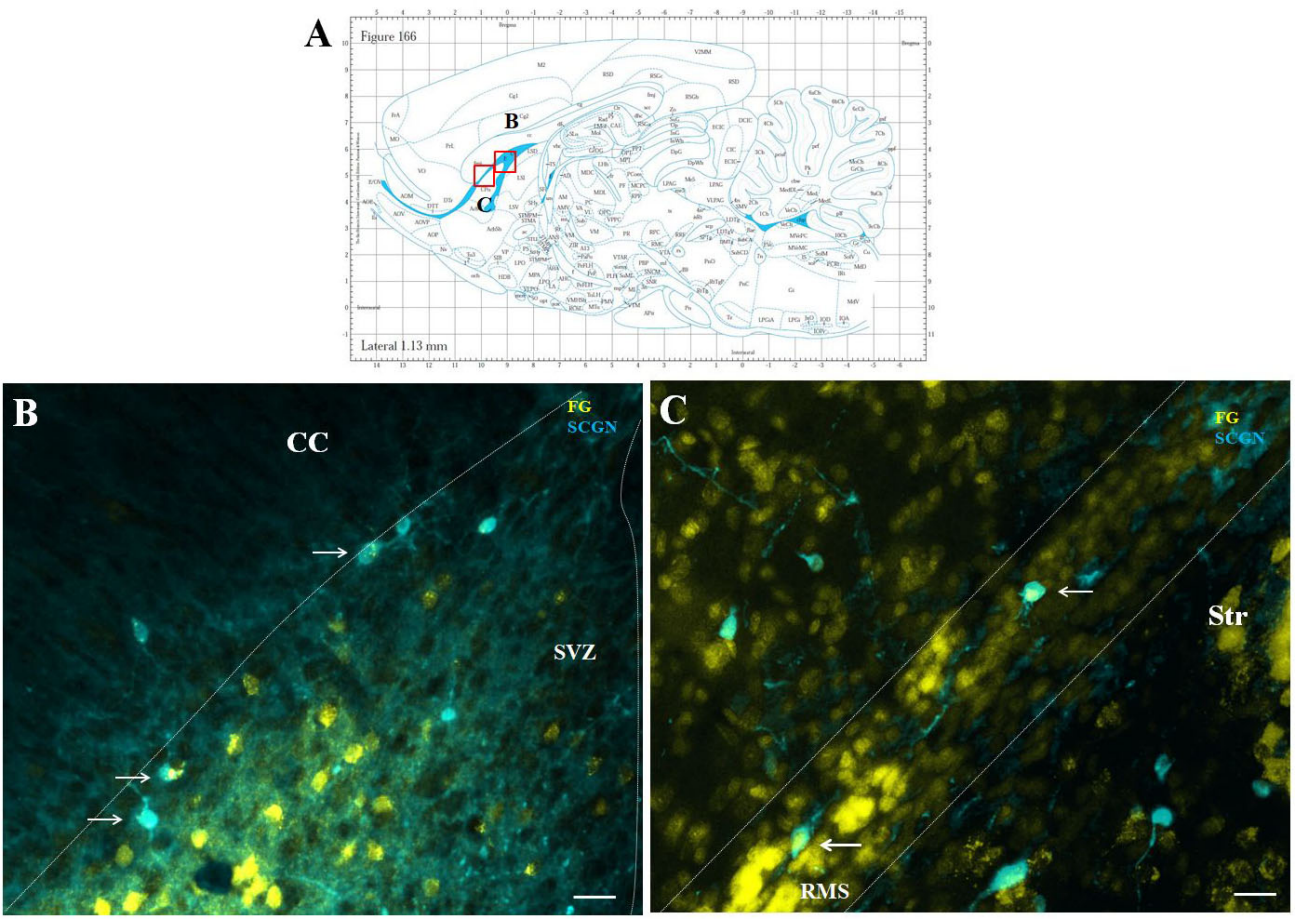
Representative confocal photomicrographs illustrating F-G-positive cells in the SVZ/RMS. Diagram of sagittal brain section from the rat brain atlas was adapted from Paxinos and Watson (2013). Red squares indicate the areas of the photomicrographs that are below **(A)**. SCGN-positive neurons (blue) co-labeled with F-G (yellow) in the SVZ **(B)** and in the vertical arm **(C)** of the RMS (indicated by arrows). The SVZ and the RMS are demarcated by dotted lines. Scale bar 25 μm. RMS, rostral migratory stream; CC, corpus callosum; SVZ, subventricular zone; Str, striatum.

Double labeling with SCGN and F-G showed co-localization in several cells of the SVZ and RMS (Figure 6). These double-labeled F-G+/SCGN+ cells were morphologically different from F-G+/nNOS + neurons. They had a round to oval morphology with short, less-developed processes. Co-labeling of F-G-labeled SCGN + neurons with a synaptic marker revealed synaptophysin-positive dots at their projection terminals in the striatum (data not shown). However, this labeling was less prominent compared to the terminals of nNOS-positive neurons.

### 3.3 Quantitative analysis of cell types in the SVZ/RMS

Quantitative analysis of the morphology of nNOS+ and SCGN + cells revealed marked differences in cell size, both within the SVZ/RMS or across cell phenotypes. The largest cells were nitrergic neurons located in the SVZ (area = 424.13 ± 129.65 μm^2^, mean diameter = 23.22 ± 1.48 μm), while nNOS + neurons in the RMS were of medium size (area = 282.13 ± 36.90 μm^2^; mean diameter = 18.93 ± 0.51). SCGN + cells were smaller than nitrergic cells, both in the SVZ (area = 177.75 ± 25.42 μm^2^; mean diameter = 15.05 ± 0.59) and RMS (area = 119.00 ± 50.84 μm^2^; mean diameter = 12.31 ± 0.60).

The quantification of cell types was performed separately in the SVZ and RMS (Table 1).

**TABLE 1:** The numbers of F-G+, nNOS+, SCGN+, F-G+/nNOS+ and F-G+/SCGN+ cells in the SVZ and in the RMS vertical arm.

Neurogenic area	FG + nNOS - SCGN-	FG – nNOS + SCGN-	FG – nNOS - SCGN+	FG + nNOS + SCGN-	FG + nNOS - SCGN+
SVZ	7.75 ± 0.59	1.88 ± 0.64	8.50 ± 0.63	6.63 ± 0.86	10.38 ± 0.91
RMS	8.00 ± 0.71	3.13 ± 0.58	8.88 ± 0.77	5.18 ± 0.98	9.75 ± 1.03

SVZ, subventricular zone; RMS, rostral migratory stream. Data are shown as mean ± SEM.

**FIGURE 7:**
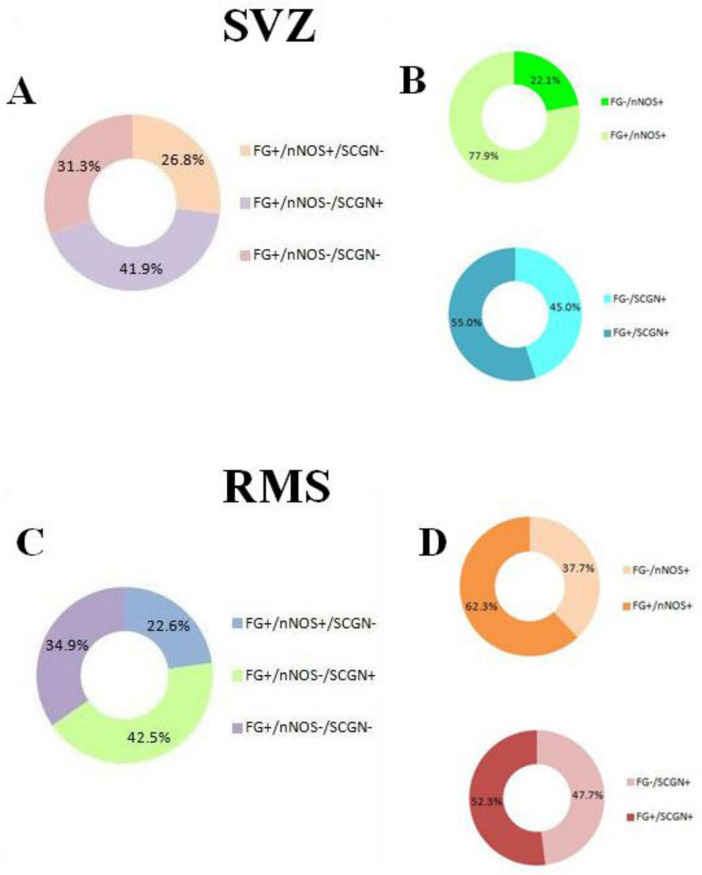
Quantitative analysis of cell types in the SVZ and RMS. **(A)** Percentage of F-G-labeled cells in the SVZ co-labeled with nNOS, SCGN or without any co-labeling. **(B)** Percentage of nNOS-labeled cells in the SVZ co-labeled with F-G vs. without F-G-labeling (green pie chart), percentage of SCGN-labeled cells in the SVZ co-labeled with F-G vs. without F-G- labeling (blue pie chart). **(C)** Percentage of F-G-labeled cells in the vertical arm of the RMS co-labeled with nNOS, SCGN or without any co-labeling. **(D)** Percentage of nNOS-labeled cells in the vertical arm of the RMS co-labeled with F-G vs. without F-G-labeling (orange pie chart), percentage of SCGN-labeled cells in the vertical arm of the RMS co-labeled with F-G vs. without F-G- labeling (red pie chart). Note that the majority of nNOS + cells and more than half of SCGN + cells were also labeled with the F-G in both areas, the SVZ and RMS **(B,D)**.

Based on the quantification of cell types (Table 1), we found that 26.8% (6.63 ± 0.86) and 41.9% (10.38 ± 0.91) of all F-G- labeled cells (24.75 ± 1.24) in the SVZ were nNOS+ and SCGN+, respectively. The remaining 31.3% (7.75 ± 0.59) of F-G-labeled cells (24.75 ± 1.24) were neither nNOS+, nor SCGN+ (Figure 7A). On the other hand, 77.9% (6.63 ± 0.86) of nNOS + cells (8.50 ± 1.31) and 55.0% (10.38 ± 0.91) of SCGN + cells (18.88 ± 0.85) in the SVZ were F-G+ and 22.1% (1.88 ± 0.64) of nNOS + cells (8.50 ± 1.31) and 45% (8.50 ± 0.63) of SCGN+ (18.88 ± 0.85) cells, in the SVZ, were F-G– (Figure 7B).

In the vertical arm of the RMS, 22.6% (5.18 ± 0.98) and 42.5% (9.75 ± 1.03) of all F-G + cells (22.93 ± 1.46) were nNOS + cells and SCGN + cells, respectively. 34.9% (8.00 ± 0.71) of F-G-labeled cells (22.93 ± 1.46) were neither nNOS+, nor SCGN+ (Figure 7C). Furthermore, 62.3% (5.18 ± 0.98) of nNOS + cells (8.31 ± 1.07) and 52.3% (9.75 ± 1.03) of SCGN + cells (18.63 ± 0.78) were F-G+, whereas 37.7% (3.13 ± 0.58) of nNOS + cells (8.31 ± 1.07) and 47.7% (8.88 ± 0.77) of SCGN + cells (18.63 ± 0.78) in the RMS were F-G– (Figure 7D).

### 3.4 F- G-positive cells outside the SVZ/RMS

After F-G administration to the striatum, F-G-positive cells were also found in distinct brain structures, such as the hippocampus, cerebellum, prefrontal cortex, substantia nigra and thalamus (Supplementary Figure 3).

**FIGURE 8:**
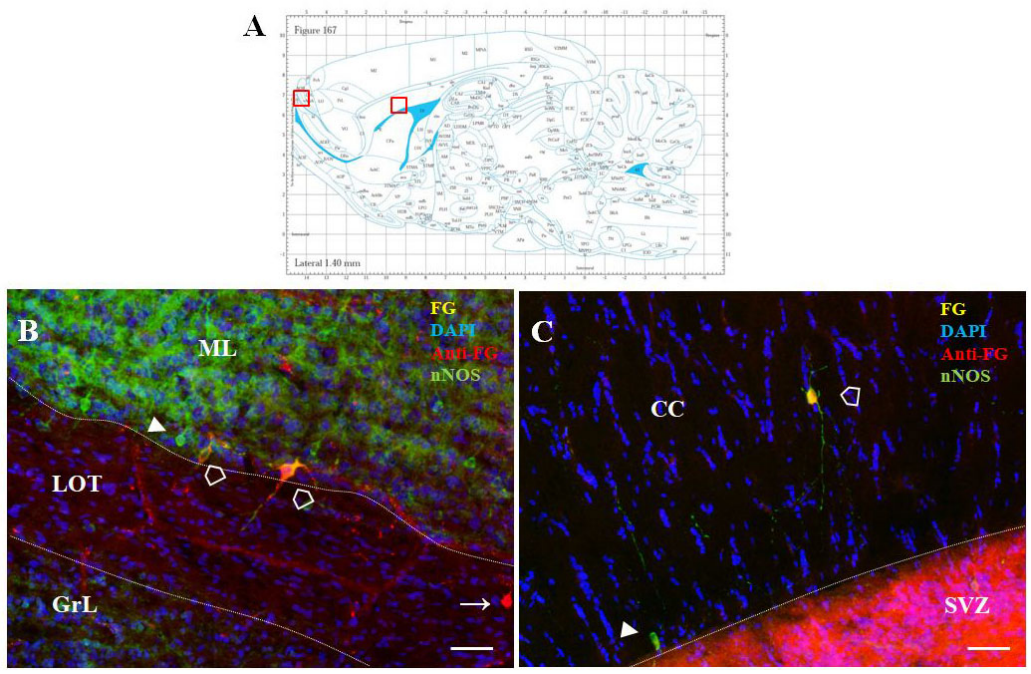
F-G-positivity in the AOB and CC. Diagram of sagittal brain section from the rat brain atlas was adapted from Paxinos and Watson (2013). Red squares indicate the areas of the photomicrographs that are below **(A)**. Distribution of F-G labeled (yellow), anti-Fluoro-Gold-positive (red) and nNOS-positive (green) cells within the accessory olfactory bulb **(B)** and the corpus callosum **(C)** of adult rat. Cell nuclei were counterstained with DAPI (blue). **(A)** Scale bar: 100 μm. Open arrowheads (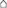): anti-F-G-positive + nNOS-positive cells; arrowheads (▶): anti-F-G-negative + nNOS-positive cells; arrows (→): anti-F-G-positive + nNOS-negative cells. SVZ, subventricular zone; CC, corpus callosum; ML, mitral cell layer; LOT, lateral olfactory tract; GrL, granular layer.

In addition, a small number of F-G-positive cells were scattered within the OB and AOB, the target structures of the SVZ/RMS cells, as well as in the structure adjacent to the SVZ/RMS, the corpus callosum (Figure 8).

Whereas in the OB only faint F-G-immunopositivity was observed, in the AOB intensely stained F-G + cells were present. These cells were embedded within the mitral cell layer of the AOB, mainly in the ventral part of this layer (Figure 8B), and only few of them were nNOS+. In addition, sparse F-G+/nNOS+ cells were also observed inside the CC, where the population of NO-producing neurons was scattered throughout its anteroposterior extent (Figure 8C).

## 4 Discussion

Adult neurogenesis in the main neurogenic area of the mammalian brain – the SVZ-RMS – is regulated by a multitude of diverse molecular factors. The principles of this regulation have been intensively studied (Li et al., 2014; Tong et al., 2014; Jurkowski et al., 2020; Niklison-Chirou et al., 2020). Several mechanisms involving diffusible signaling molecules, such as neurotransmitters (Banasr et al., 2003; Tong et al., 2014), neuropeptides (Agasse et al., 2008; Decressac et al., 2009), and growth factors (Palmer et al., 2000; Bath and Lee, 2010), have been proposed to regulate SVZ neurogenesis and its effects on olfactory tuning and plasticity.

Recent important studies have demonstrated that adult neurogenesis in the SVZ can also be modulated by neural circuit regulation (Paez-Gonzalez et al., 2014; Naffaa et al., 2023). It was revealed that a small population of mature ChAT + neurons is involved in a neural circuit that controls the activation of quiescent neural stem cells in the SVZ (Paez-Gonzalez et al., 2014; Naffaa et al., 2021). While Chat + neurons have been found directly in the subependymal space adjacent to the lateral ventricle (Paez-Gonzalez et al., 2014), the nitrergic and SCGN-positive neuronal populations described in this study are distributed throughout the entire extent of the SVZ. Moreover, both of these neuron types, with their characteristic distribution patterns, are also present in the RMS. To our knowledge, however, ChAT^+^ neurons have only been described in the SVZ. Additionally, our preliminary data did not show any F-G labeling in ChAT + neurons in the SVZ following F-G administration to the striatum (data not shown). Therefore, we suggest that nitrergic and SCGN-positive neurons in the SVZ represent distinct neuronal populations different from the previously described cholinergic neurons.

Evidence for the presence of neurons located directly in the neurogenic region SVZ-RMS (Raceková et al., 2005; Blasko et al., 2013; Hanics et al., 2017) suggests that these neurons could also be involved in the regulation of neurogenesis in the SVZ-RMS. In the RMS, two cell populations have been identified: NO-producing neurons (Raceková et al., 2005) and neurons expressing SCGN (Hanics et al., 2017). We have shown that NO-producing neurons are located predominantly in the caudal part of the RMS, i.e., in the part directly emerging from the SVZ and continuing between the striatum and CC, referred to as the RMS vertical arm (Raceková et al., 2005). These neurons exhibit the typical morphological characteristics of mature neurons, including well-developed varicose processes. Some of these processes penetrate deeply into the adjacent brain structures, particularly the CC and striatum (Raceková et al., 2005, 2003). Later, we provided immunohistochemical evidence that nitrergic neurons of the RMS form synaptic connections (Blasko et al., 2013), indicating their functionality. Regarding the role of NO, it has been demonstrated that NO acts as an important regulator of cell proliferation in the adult brain (Packer et al., 2003; Gutiérrez-Mecinas et al., 2007). Besides nitrergic neurons, another population of neurons expressing secretagogin has been identified in the adult rodent RMS (Hanics et al., 2017). These neurons facilitate neuroblast migration through the RMS by externalizing MMP-2 (Hanics et al., 2017). The significance of both types of neurons in the regulation of adult neurogenesis indicates that their functioning is essential for the normal course of neurogenesis. Therefore, it was crucial to reveal the connectivity of these neurons located in the SVZ/RMS region. To this end, we injected retrograde tracer F-G into the striatum of adult rats. Based on fluorescence microscopic analysis of sagittal brain sections, we found F-G-labeled cells in the SVZ and RMS. Additionally, we found F-G labeled cells in several brain structures including the hippocampus, cerebellum, prefrontal cortex, substantia nigra and thalamus that are known to be connected to the striatum (Lanciego et al., 2012; Hunnicutt et al., 2016; Bostan and Strick, 2018; Haber et al., 2021; Contreras-López et al., 2023). Surprisingly, we also detected F-G-labeled cells within the CC. These F-G-positive cells in the CC showed nNOS positivity. Although it is known that the CC contains nitrergic neurons (Barbaresi et al., 2020), to the best of our knowledge, its connection with the striatum has not been demonstrated.

In the SVZ and the RMS, we identified F-G-labeled cells as nitrergic neurons or SCGN-producing cells. The presence of these F-G-labeled nNOS/SCGN-positive neurons in the SVZ-RMS, confirms a neuronal connection between the striatum and the SVZ-RMS. Moreover, our results, based on co-labeling with a synaptic marker revealed synaptic connectivity of nNOS+ and SCGN + neurons of SVZ/RMS with the striatum. This suggests the existence of a neural circuit that may be involved in the regulation of neurogenesis.

Interestingly, synaptophysin labeling reflected the morphological differences between nNOS+ and SCGN + cells in the SVZ and RMS. Large nitrergic neurons in the SVZ, as well as intermediate-sized nitrergic neurons in the RMS, showed strong synaptophysin expression, which may indicate a high level of synaptic integration and NO production. In contrast, synaptophysin labeling was less prominent on the terminals of the smaller SCGN + cells, suggesting they have distinct roles that are less dependent on extensive synaptic connectivity.

The connection between the striatum and nitrergic neurons of the SVZ/RMS could serve several physiological and functional purposes. Under physiological conditions NO plays a critical role in modulating the proliferation, migration, and differentiation of progenitor cells (Estrada and Murillo-Carretero, 2005). Studies investigating the role of NO in neurogenesis considered cells located at the periphery of the SVZ/RMS as the source of NO (Moreno-López et al., 2000; Packer et al., 2003). Our previous findings revealed the presence of NO-producing neurons not only in the vicinity of the neurogenic regions but directly inside the SVZ/RMS (Raceková et al., 2005, 2003). The novel finding of the present study is the morphological confirmation their connection with the striatum.

It has been found that NO-producing GABAergic striatal neurons project into the SVZ (Moreno-López et al., 2000). These striatal GABAergic neurons have been shown to reach out neuroblasts and neural progenitor cells in the SVZ (Young et al., 2014). Depolarization of striatal neurons leads to GABA_*A*_R-mediated calcium activity in SVZ cells, which is known to regulate progenitor cell proliferation (Young et al., 2014). This suggests that nitrergic/GABAergic neurons from the striatum may influence neurogenesis through these direct projections, with NO acting as a critical modulator in this process. We hypothesize that NO-producing neurons located in the SVZ and RMS, which send their processes to the striatum as demonstrated here, may be part of a neural circuit involving striatal nitrergic/GABAergic neurons that regulates postnatal neurogenesis in the SVZ. Theoretically, these NO-producing neurons in the SVZ-RMS may also have reciprocal effects on striatal circuits, potentially forming a feedback loop that balances neurogenesis. However, further studies are needed to confirm these hypotheses.

Secretagogin, a calcium-binding protein, plays an important role in regulating neural progenitor cell behavior in the SVZ, including aspects of migration, survival, and differentiation (Alpár et al., 2012). While the functional significance and precise role of the connection between SCGN-positive neurons in the SVZ/RMS and the striatum are not yet fully understood, there are findings that suggest a potential link. SCGN-producing neurons in the SVZ may contribute to the regulation of neurogenesis, particularly by supporting the migration of newly generated neurons to other brain regions, including the striatum (Ming and Song, 2011; Alpár et al., 2012). In the RMS, SCGN-expressing neurons form a scaffold. These neurons can externalize the enzyme matrix metalloprotease-2 (MMP-2) that loose extracellular matrix and thus facilitate the migration of neuroblasts in the RMS (Hanics et al., 2017). Regarding SCGN-producing neurons in the striatum, it has been shown that they are integral to the functional organization of striatal circuits (Garas et al., 2016). They help modulate motor control and rewards processing. Furthermore, some SCGN-producing neurons, specifically calretinin-producing neurons, are located in the subventricular region and at the rostral pole of the striatum (Kosaka et al., 2017), i.e., near the ventral margin of the RMS vertical arm. These findings suggest that the neural circuit involving SCGN-producing neurons in the SVZ and the striatum may play a role in various aspects of neuroplasticity, sensorimotor integration, and neurogenesis.

Taken together, the connection between the SVZ-RMS and other brain structures provides new insights into the complexity of adult neurogenesis, a process much more intricate than originally believed. Our findings contribute to a better understanding of adult neurogenesis, and could be useful in the development of new therapeutic strategies that enable targeted, localized delivery of therapeutic agents aimed at neuronal replacement following various types of injury or in the treatment of neurodegenerative diseases. Further investigation using advanced methods is required to elucidate the SVZ/RMS-striatum neuronal circuit and its role in the regulation of SVZ-RMS neurogenesis.

## Data Availability

The raw data supporting the conclusions of this article will be made available by the authors, without undue reservation.
