# Supplementary material for: Morphological evidence for neuronal connections between the olfactory neurogenic region and the striatum in adult rats
Source: Front Neural Circuits. 2025 Sep 17;19:1605961. doi: 10.3389/fncir.2025.1605961 (PMC12484237; doi:10.3389/fncir.2025.1605961)
Supplement: Supplementary file 1 [file Table_1.docx]

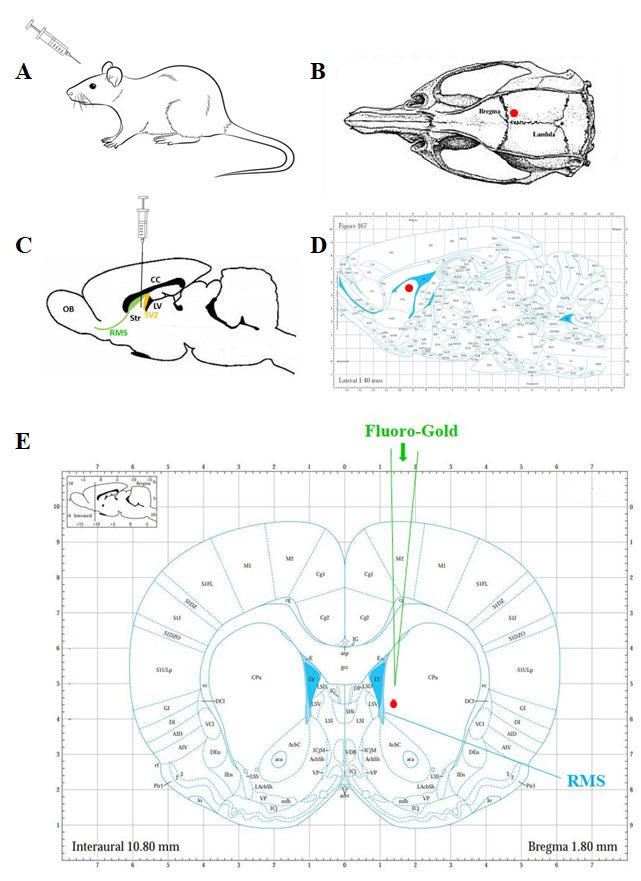


**Supplementary figure 1:** **Experimental design.** **Stereotactic injection of the Fluoro-Gold into the striatum**; **(A)** F-G injection principle, **(B)** Horizontal, **(C, D)** sagittal and **(E)** coronal schematic drawings of injection site. The red mark indicates the site of intracerebral administration of F-G. F-G was administered to the striatal region, near the rostral migratory stream. OB – olfactory bulb, SVZ – subventricular zone, RMS – rostral migratory stream, Str – striatum, CC – corpus callosum, LV – lateral ventricle. **(D, E)** Coronal brain sections from the rat brain atlas were adapted from Paxinos and Watson (Paxinos and Watson, 2013).
