# Supplementary material for: Morphological evidence for neuronal connections between the olfactory neurogenic region and the striatum in adult rats
Source: Front Neural Circuits. 2025 Sep 17;19:1605961. doi: 10.3389/fncir.2025.1605961 (PMC12484237; doi:10.3389/fncir.2025.1605961)
Supplement: Supplementary file 2 [file Table_2.docx]

**
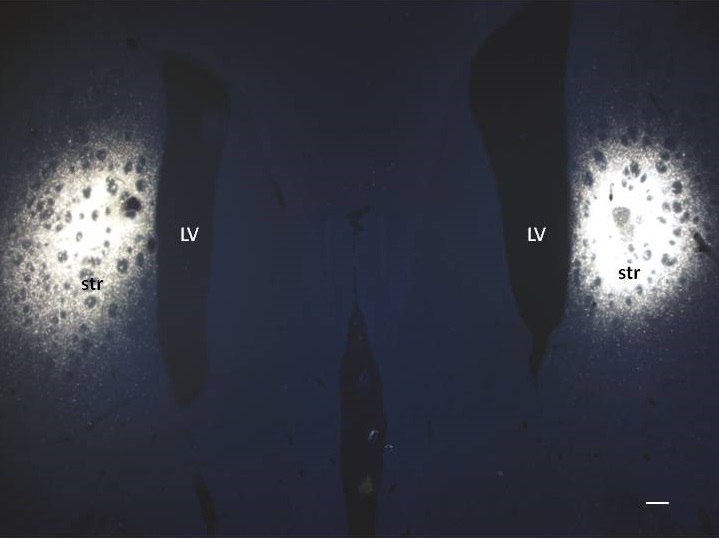
**

**Supplementary figure 2: Bilateral injection of F-G into the striatum.** Photomicrograph of the coronal section after bilateral injection of F-G to the striatum. Note, that the injection site was precisely localized, the injected area was well demarcated and symmetrical in both hemispheres. Scale bar 100 µm. LV – lateral ventricle, str - striatum
