# Supplementary material for: Morphological evidence for neuronal connections between the olfactory neurogenic region and the striatum in adult rats
Source: Front Neural Circuits. 2025 Sep 17;19:1605961. doi: 10.3389/fncir.2025.1605961 (PMC12484237; doi:10.3389/fncir.2025.1605961)
Supplement: Supplementary file 3 [file Table_3.docx]

**
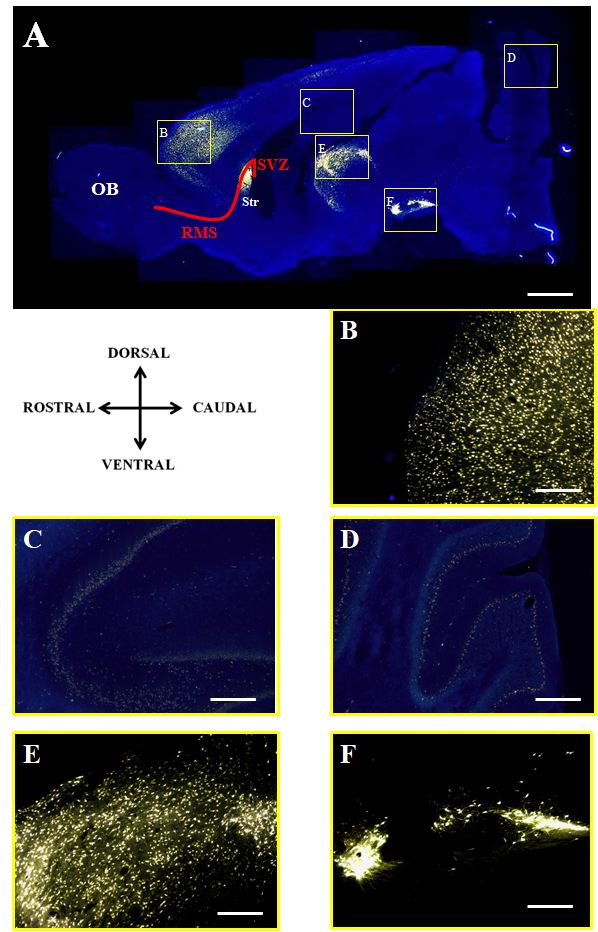
**

**Supplementary figure 3: F-G-positivity in the rat brain after administration into the striatum.** (A) Sagittal section of the rat brain showing the distribution of F-G-positive cells. Boxed areas show magnifications of (B) prefrontal cortex, (C) hippocampus, (D) cerebellum, (E) thalamus and (F) substantia nigra. Scale bar (A) 2 mm, (B-F) 500 µm. SVZ – subventricular zone, RMS – rostral migratory stream, OB – olfactory bulb, Str – striatum.
